# The Influence of Instruction on Public Health

**Published:** 1873-07

**Authors:** 


### Editorial.

### The Influence of Instruction on Public Health.

We have often called attention to the fact that the public are in great need of more light on the subject of Sanitary Science.

The laws which govern the accumulation of wealth are given a prominent position in the curriculum of our schools and colleges, while less attention is paid to the maintainance of the public health than is given to the languages and institutions of the antediluvians.

The popular mind is not prepared to receive instruction on purely medical topics, and this instruction would not be necessary for them if they were so prepared. What is needed by the populace is a knowledge of the sanitary and hygienic rules to be observed in the preservation of health.

It has been noticed by all observing minds that where general instruction was the most diffused, there the mortality was the least. Misery and squalor disappear before the onward march of popular education.

We do not know that this matter has been made the subject of statistical observations in any other country than France, and we present our readers with a brief resume of the facts derived from these observations taken from our valuable contemporary, *The Doctor*, (London):

"Even in 1858 Meher had demonstrated that those French departments in which instruction was diffused were in general those which presented the fewest deaths, and had found that the map of France, colored differently according to the amount of mortality, corresponded in the depth of its tint with that constructed by Dupin with respect to instruction. Dr. Mesnil undertook his researches by consulting the books of *recrutement* from 1841 to 1849, the number of deaths from 59 to 60, the proportion of exemptions from shortness of stature from 1837 to 1849, and from 1850 to 1859. He came to the following conclusion:

"1. The departments where the proportion of ignorant persons is greatest are also those where the mean of life is shortest and the stature the least. 2. Those departments in which instruction is most spread abroad (excepting the departments where there is the greatest agglomeration of persons) are the ones in which the mean of life is greatest and the stature most elevated. For instance, in the department of the Haute Marne, which occupies No. 5 on the instruction chart, there is a mean of life of 41.6, and a stature of 1.66 metres, whilst in the department of Finisterre, which is No. 82 on the instruction chart, the average of life is 29.2, and the mean height 1.62 metres

"The departments where instruction is sparingly diffused present the greatest mortality, and those deprtments which occupy the first ten numbers on the educational chart have always been very healthy. The departments less educated present also the greater number of exemptions from military service. Thus Dordogne, which is 81 on the chart of instruction, has No. 88 on the map of military exemptions. The exemptions made from want of sufficient stature correspond also to the grade of instruction. Thus the department of Cotes du Nord has 82 as its number in the education map, and a like number on its map for military exemption for shortness of stature. The department of Sardes of the Haute Loire and Dordogne also have No. 81 in both of these points. The provinces which have the greatest diffusion of instruction are those which the greatest increase in numbers. Thus, according to Leroy-Beaulieu, in thirty years France (from 1836–66) had an annual mean of 0.44 increment in population. It is impossible to separate these three words: ignorance, misery, and mortality. That population which the schoolmaster finds indifferent to the benefits of education, becomes through its ignorance rebellious to the application of new discoveries in arts and industry, and to the observance of hygienic rules."

It will thus be seen that in direct proportion to the amount of instruction received the mortality decreases and physical developement is increased.

We are justly proud of our common school system in America, and its influence upon the masses is marked; but could not that influence be exerted with a greater degree of force if in our system of popular education some measure of attention was directed toward the care of the body and the mind.

In the expectation of an epidemic of cholera our people are aroused to the fact that the hygienic surroundings are in a sadly neglected condition, and our newspapers teem with directions for improving them. Our boards of health are soundly abused from all sides, and perhaps with some cause, for the bad condition of things; but their hands are virtually chained. With the present condition of public information on the subject of hygiene, were they to carry out to their full extent the improvements neeeded, their efforts would be met with a still greater storm of abuse, and the cry of excessive and useless taxation would be long and loud.

Until the public are educated up to a higher standard of information regarding the laws of health, we must despair of any attempt to improve the sanitary condition of our cities and towns. The day is, however, approaching, we feel confident, when the physician will be looked upon as the guardian of health as well as a minister to disease, when his duty shall be to teach the public how to preserve health as well as correct conditions of disease incurred by non-observance of the laws of nature.

As an evidence of this fact we are happy to notice the efforts put forth by the American Public Health Association to instruct the public how to guard against the approach of an epidemic of cholera. We hope their efforts will not cease here, but that through the medium of the press they will endeavor to diffuse that information which is so much needed regarding the care of the healthy as well as the deceased.

